# Considering the full care pathway in regional variation in paediatric otitis media treatment in the Netherlands: an observational study

**DOI:** 10.1136/bmjopen-2025-101692

**Published:** 2025-08-19

**Authors:** Vera de Weerdt, Christel van Dijk, Jako Burgers, Kati Gaspar, Karin Hek, Raphael J Hemler, Sjoerd Repping, Robert A Verheij, Hanna C Willems, Eric J E van der Hijden, Xander Koolman

**Affiliations:** 1 Health Economics, Vrije Universiteit, Amsterdam, the Netherlands; 2 Amsterdam University Medical Centers, Amsterdam, the Netherlands; 3 National Healthcare Institute Diemen Netherlands, Diemen, the Netherlands; 4 Nederlands Huisartsen Genootschap, Utrecht, Utrecht, the Netherlands; 5 Maastricht University CAPHRI School for Public Health and Primary Care, Maastricht, the Netherlands; 6 CPB Netherlands Bureau for Economic Policy Analysis, the Hague, the Netherlands; 7 NIVEL, Netherlands Institute for Health Services Research, Utrecht, the Netherlands; 8 Otholaringology, Gelre Hospitals, Apeldoorn, the Netherlands; 9 University of Amsterdam, Amsterdam, the Netherlands; 10 NIVEL, Utrecht, the Netherlands; 11 Tilburg University Tilburg School of Social and Behavioral Sciences, Tilburg, the Netherlands; 12 Department of Internal Medicine and Geriatrics, University of Amsterdam, Amsterdam, the Netherlands; 13 Zilveren Kruis Health Insurance, Leiden, the Netherlands

**Keywords:** Quality Improvement, Paediatric otolaryngology, Paediatric otolaryngology, Primary Care

## Abstract

**Abstract:**

**Objective:**

Ventilation tube insertion for paediatric otitis media (POM), including acute otitis media (AOM) and otitis media with effusion (OME), has been signalled in the past for potential unwarranted treatment variation. Quality improvement initiatives, like Audit & Feedback (A&F), often ignore the care pathway when identifying such variation, possibly overestimating variation at a specific care step. To gain more insight into the effect of prior care steps, this study examined (1) the degree of regional variation in each step of the care pathway (general practitioner (GP) contacts, referrals and surgeries) and (2) investigated the effect of adjusting for prior care steps.

**Design:**

Observational study using general practice electronic health record data linked to specialist claims data.

**Participants:**

272 790 children ≤12 years with and without POM registered in 320 GP practices between 2017 and 2018.

**Primary and secondary outcomes:**

Using multilevel logistic regression, the degree of regional variation in each step of the POM care pathway was assessed by calculating the coefficient of variation (CV).

The effect of adjusting for prior care steps was determined by estimating correlations between subsequent care steps and analysing the impact on the CV.

**Results:**

Regional variation in POM treatment was larger in each subsequent step in the care pathway (CV POM GP contacts 0.110; referral 0.179; surgery 0.239). In regions with a higher proportion of children with frequent AOM/persistent OME, referral rates were higher (POM: OR: 1.06; 95% CI: 1.02 to 1.11) and surgical rates were higher (for OME only: OR: 1.08; 95% CI: 1.02 to 1.15). Regional variation in referrals and surgery decreased after adjusting for the regional frequent AOM/persistent OME rate (CV referrals POM 0.103 vs 0.128; CV surgery OME 0.047 vs 0.059).

**Conclusions:**

Regional variation is observed in GP contact rates for POM and is larger in referrals and surgeries. Adjusting for the proportion of frequent AOM/persistent OME significantly reduces regional variation in POM treatment. Future A&F should adjust for prior care processes and develop tailored interventions for quality improvement.

**Strengths and limitations of this study**

- The study examined the degree of regional variation in each step of the care pathway using general practice electronic health record data linked to specialist claims data.
- By adjusting for the prior care steps, regional variation at the subsequent step in the care pathway could be assessed more precisely.
- Due to the use of electronic health record data, limited information was available on disease severity.
- Variation was only assessed at the regional level. Variation at the general practitioner (GP) practice or hospital level could provide deeper understanding of the role of GPs versus specialists.

## Background

 Ventilation tube insertion (VTI) for paediatric otitis media (POM), including acute otitis media (AOM) and otitis media with effusion (OME), is one of the most frequently performed paediatric procedures in hospitals worldwide.^1^,^3^ In the past, this surgery was signalled for potential unwarranted treatment variation, and it became a topic of deimplementation initiatives.^4^,^6^ While VTI surgery benefits some children with POM, it was increasingly performed on those unlikely to benefit.^1^ Quality improvement initiatives, such as Audit & Feedback (A&F), often focus on the reduction of variation in specialist treatment, without accounting for prior care processes like general practitioner (GP) contacts and GP referrals.^7 8^

The Dutch healthcare system is characterised by a gatekeeper model with obliged registration in GP practice. In this model, GPs have a gatekeeping role for specialist care. Patients can first consult a GP for diagnosis and treatment. GP consultations are reimbursed without copayment by basic health coverage.^9^ When necessary, GPs refer to specialist care for diagnosis and treatment. Thus, parents of children with POM initially contact their GP. National guidelines are available supporting GPs and Ear–Nose–Throat (ENT) specialists in decision making. These include criteria for referral to the ENT specialist for diagnosis and treatment. Within this system, medical treatment variation can emerge in multiple stages of the POM care pathway: (1) in parents’ decisions regarding GP consultation, (2) in GPs’ decisions regarding referral and (3) in differences in ENT specialists’ decisions, such as choosing surgical versus non-surgical treatment (see box 1 for more information on POM care in the Netherlands).

Box 1 **Paediatric otitis media care in the Netherlands**

Children with otitis media commonly present to GPs, where they are typically diagnosed with AOM or OME. Since most AOM episodes resolve spontaneously within days, the Dutch GP guidelines (NHG) recommend watchful waiting without antibiotic treatment.^23^ A referral for AOM is recommended in cases with alarm symptoms, such as suspected mastoiditis, in cases where oral antibiotics fail to improve symptoms, or for children with frequent recurrent infections (≥3 episodes within 6 months or ≥4 episodes within 1 year). Dutch ENT guidelines recommend considering surgical treatment with ventilation tube insertion for frequently recurring AOM, following the same definition of GPs (≥3 episodes within 6 months or ≥4 episodes within 1 year).^24^

For OME, the Dutch GP guideline recommends watchful waiting and does not recommend any pharmacological interventions (NHG).^17^ Referral for surgical intervention may be considered if symptoms persist for more than 3 months, if symptoms are associated with significant impairment of the child’s functioning or development, and/or if the hearing loss exceeds 26 dB in the better ear. The Dutch ENT guideline recommends considering surgical treatment with ventilation tube insertion for persistent OME in children under 4 years if their speech-language development is at risk or if they experience significant hearing loss.^18^ For children aged 4 years or older, ventilation tube insertion is recommended if hearing loss causes substantial difficulties, and their hearing loss is >20 decibel.

AOM, acute otitis media; OME, otitis media with effusion; GP, general practitioners; ENT, Ear–Nose–Throat.

Each actor—patients, GPs and ENT specialists—plays a distinct role in the care pathway, either mitigating or amplifying unwarranted treatment variation. Negative associations between steps in the care pathway suggest a mitigating role, while positive associations point to an amplifying role in creating treatment variation. Likewise, negative associations indicate independent clinical decision-making by actors, whereas positive associations suggest a positive feedback loop. In this positive feedback loop, the performance of a referral or VTI surgery gives feedback to the previous actor that decision making at their level, that is, to contact the GP or to refer, was appropriate, while the clinical decision may not have been aligned with guidelines.

A&F is one of the most frequently used interventions to motivate professionals to reduce unwarranted variation and to adopt consistent, evidence-based practices.^10^,^12^ A&F is most effective when it targets aspects of clinical practice that healthcare professionals can directly influence.^13^,^15^ Therefore, when designing A&F, it is important to target the right professional and to account for the role of patients and other professionals in treatment variation.

This study examines (1) the degree of regional variation in POM in each step of the POM care pathway (GP contacts, referrals and surgeries) and (2) the effect of adjusting for prior care steps when identifying POM treatment variation.

## Methods

### Data source

This observational study used routinely collected healthcare data, derived from general practice electronic health records (EHRs) linked to medical claims data from specialist care. Data were linked on patient level by a trusted third party (ZorgTTP) using pseudonymised social security numbers. EHR data were derived from GP practices that contributed data to Nivel Primary Care Database (Nivel-PCD). Nivel-PCD encompasses routinely recorded data from approximately 10% of Dutch GP practices and about 10% of the Dutch population.^16^ The database includes longitudinal information on GP consultations, diagnoses, treatments, drug prescriptions, and patient age and sex. Diagnoses are recorded using the International Classification of Primary Care (ICPC) V.1. Prescriptions are recorded using the Anatomical Therapeutic Chemical classification system.^17^

Claims data were provided to the Dutch National Healthcare Institute (NHCI, in Dutch: Zorginstituut Nederland) by Vektis, the business intelligence centre of Dutch health insurers. Vektis receives claims data from all Dutch health insurers, including medical claims for the entire Dutch insured population (~99% of the entire Dutch population) under the Health Insurance Act. This includes claims for medical specialist care, general practice care, allied healthcare and outpatient pharmaceutical care. The database has 100% national coverage and includes all care covered by the basic health insurance scheme. Claims data include information on age, gender, 4-digit ZIP code and various other plan holder details. Medical specialist care is predominantly remunerated via a Diagnosis Treatment Combination system, which is comparable to a Diagnosis Related Group (DRG) structure, but is specifically developed for the Netherlands.

### Patient and public involvement

Patients and/or the public were not involved in the design, or conduct, or reporting or dissemination plans of this research.

### Study population

Data were included for patients who were registered in general practices participating in the Nivel-PCD and aged 12 years or younger, as the incidence of POM is highest in this age category.^16 18 19^ Patients diagnosed with AOM or OME in 2017 or 2018 were identified using ICPC V.1 codes H71 for AOM and H72 for OME. We studied the combined POM population and the separate AOM and OME population, as indications for referral for AOM and OME differ, but the distinction between AOM and OME in GP practice is not always clear. The study period of 2017–2018 was selected as the most recent timeframe allowing a follow-up of 1 year and 3 months while avoiding potential bias from the COVID-19 pandemic period.

Inclusion criteria were (1) no prior diagnosis of AOM, OME or ear pain (ICPC code H01) in the year preceding the POM diagnosis, (2) no VTI surgery in the 2 years before the diagnosis, (3) complete demographic data—including age, gender, neighbourhood socioeconomic status (SES) and level of urbanicity, (4) listed within the same general practice within Nivel-PCD during the year of diagnosis, the preceding year and the following year, (5) patients’ GP practice provided complete EHR data to the Nivel-PCD and (6) patients’ health insurer provided a complete set of claims data for medical specialist care in the 2 years prior to diagnosis, the year of diagnosis and the 2 years following diagnosis. The final study population comprised 272 790 children≤12 years in 320 GP practices, of whom 13 117 children with AOM, 3972 children with OME and 15 727 children with POM over the years 2017 and 2018. This constitutes 6% of the Dutch population up to 12 years and 7% of the GP practices.^17 20^

### Regions

Treatment variation was analysed at the regional level, as patient populations across regions are more comparable in terms of relevant characteristics than patient populations of individual healthcare providers.^12^ As a result, disease incidence, prevalence and severity are more consistent across regions. Regional variation is therefore less likely to reflect differences in patient need and more likely to indicate unwarranted variation.^21^ All children were assigned to a region, based on the hospital to which their GPs referred patients with POM most frequently. Some GP practices did not refer any patients for POM during the study period. Children in these practices were assigned to the hospital to which a neighbouring GP practice (identified on the basis of 4-digit ZIP codes) referred to most frequently. This resulted in 66 distinct regions, which were used as the unit of analysis for regional variation. The number of GP practices per region varied between 1 and 19, with an average of five practices per region. The minimum number of patients with AOM per region was 4, the maximum number of patients per region was 651 and the mean number of patients per region was 193. The minimum number of patients with OME per region was 3, the maximum number was 198, and the mean was 59.

### Study outcome: regional variation

**Figure 1:**
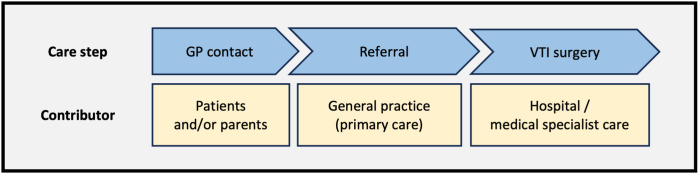
Care pathway paediatric otitis media. GP, general practitioner; VTI, ventilation tube insertion.

Regional variation in the treatment of POM was analysed at different stages of the care pathway: (1) patient-initiated GP contacts for POM, (2) referral for POM, and (3) VTI surgery for POM (figure 1).

#### Patient-initiated GP contacts

GP contacts were defined as having an ICPC code recorded for POM in general practice in 2017 or 2018 and no registered ICPC code in the previous year. This variable reflects the choice of patients and/or parents to consult their GP for POM.

#### Referral

Referrals for POM were identified based on a registered Hospital Visit with DRG code 13 (AOM, OME or tube dysfunction) in ENT care occurring within 1 year and 3 months after a POM diagnosis in general practice.

GP referrals may or may not result in a hospital visit, as patients and/or parents can choose not to consult the specialist. Since data on actual GP referrals were unavailable, we used hospital visits as a proxy indicator of GP referrals in the care pathway. This measure may likely underestimate the actual number of GP referrals. Approximately 86% of referrals in the adult population typically result in hospital visits.^22^ This rate is expected to be higher in children, as those in the basic health insurance scheme for children under 18 years old are exempted from co-payments.

#### Ventilation tube insertion

VTI surgery was identified by the presence of a claims code for a surgical procedure for VTI (code 031802) in ENT care in the year and 3 months following a POM diagnosis in general practice. This reflects the role of ENT specialists in performing surgery.

### Factor possibly explaining regional variation: frequent AOM and persistent OME

Regional variation at the level of referral or/and VTI surgery may be explained by variation in the percentage of frequent AOM and persistent OME in a region. Based on Dutch GP-guideline and ENT guidelines, frequent AOM was defined as patients in GP practices with ≥3 recorded care episodes within 6 months or ≥4 recorded care episodes within 1 year in GP practice. Frequent AOM is an indication for referral and VTI surgery for AOM according to Dutch guidelines. For OME, persistent OME was defined as patients with a care episode registered in GP practice lasting ≥3 months.^23^,^25^ Persistent OME is an indication for referral and VTI surgery for OME according to Dutch guidelines. In the combined POM population, patients were classified as frequent AOM/persistent OME if either criterion was met.

Episodes of care were identified based on recorded diagnoses of AOM, OME or ear pain (ICPC codes H01, H71, H72) in GP EHRs, including diagnoses recorded during consultations, for (recurrent) prescriptions and those based on information GPs received from other healthcare providers. Contact diagnoses were grouped into episodes of care using a contact-free interval of 28 days, meaning a subsequent diagnosis was considered part of the same episode if it occurred within 28 days of the previous one.^26^ Given that POM is frequently coded as ear pain (ICPC code H01) in GP practices, this code was included in episode construction. 

### Potential confounders

Numerous risk factors contribute to the incidence of POM, including patient age, gender, SES, genetic predisposition, atopy, presence of older siblings, daycare attendance, seasonal variations, ethnicity, air pollution and more.^27^,^31^ In this study, data were available to adjust for the following case-mix variables: patient age; gender; SES status on neighbourhood level; urbanicity (as a proxy for air quality) and comorbidity with asthma/hay fever.

Age was included as a categorical variable with four groups: 0–1 years, 2–3 years, 4–5 years and 6–12 years, reflecting the varying incidence of AOM and OME across age groups.^16 19^ Gender was included as a binary variable. Neighbourhood SES was measured using SES-WOA scores, which indicate the status of a neighbourhood relative to other neighbourhoods.^32^ This score is composed of several characteristics of inhabitants: education, income and position on the labour market, and is divided into quartiles for analysis (with a higher quartile indicating a higher SES). Urbanisation was included as a relative score with five categories, based on the density of addresses (high to low) within the patient’s ZIP code area.^33^ Comorbidity with asthma or hay fever was based on episodes for asthma (ICPC-code R96) or hay fever (R97) in general practice.^26^ In our data set, information on comorbidities was only available for the group of children with POM. In analyses on children without POM as the denominator, comorbidities could not be adjusted for.

### Analyses

All analyses were conducted separately for children with AOM, OME and the combined POM population. To enhance statistical power, the 2017 and 2018 cohorts were merged in the analyses. Statistical analyses were performed using SAS software, V.8.3.

#### Regional variation

The degree of regional variation in POM treatment was determined by calculating the coefficient of variation (CV) for three outcomes: (1) patient-initiated GP contacts, (2) referral and (3) VTI surgery. The CV was calculated from the regional probabilities for each outcome, estimated using multilevel logistic regression analysis (random intercept on regional level).^34^ A higher CV indicates greater relative variability, reflecting more treatment variation, while a lower CV suggests less relative variability. The denominator of these regressions included children up to 12 years old registered at GP practices, regardless of their diagnostic status (ie, children with and without POM). Adjustments were made for patient age, gender, neighbourhood SES and urbanicity.

To determine whether regions with a small number of children affected the results, sensitivity analyses were performed including regions with at least 500, 1000 and 2000 children. Furthermore, S-curve figures with point estimates and CIs, using a ±1.39*SE to ensure statistical difference, were developed to visually represent regional variation for each outcome.^35^

#### Effect of adjusting for the care pathway in evaluation of OM treatment variation

To assess the effect of adjusting for prior care steps (including factors potentially explaining variation: frequent AOM and persistent OME), additional multilevel logistic regression analyses were conducted with as outcome referral and VTI surgery (table 1). The outcomes referral and VTI surgery were measured at the individual level and the preceding step was measured at the regional level as a percentage. This shows whether a higher-than-expected probability (based on case-mix) of a preceding care step leads to a lower or higher probability of the subsequent care step.^8^

**Table 1:** Model specification* for examining correlation between sequential steps in the care pathway

Model	Sequential care step (dependent variable)†	Preceding care step (confounder included as variable of interest)‡	Denominator used in model
1	Referral	% of children with a patient-initiated *GP contact* for POM per region	Children with diagnosis of POM in GP practice
		% of children with *frequent AOM/persistent OME* per region	
2	Ventilation tube insertion surgery	% of children with a patient-initiated *GP contact* for POM per region	Children with referral for POM
		% of children with a *referral* for POM per region	
		% of children with *frequent AOM/persistent OME* per region	

* All models were adjusted for case-mix factors (age, gender, SES, urbanicity, asthma and hay fever) and all other preceding care steps. Results reflect only the confounder of interest.

† Measured at the individual level.

‡ Measured at the regional level.

AOM, acute otitis media; GP, general practitioner; OME, otitis media with effusion; POM, paediatric otitis media; SES, socioeconomic status.

The regional probabilities for patient-initiated GP contacts were derived from the analyses as described in the paragraph ‘regional variation’ and were thus based on analyses using the denominator ‘children up to 12 years old’. The analyses for the outcome referrals and frequent AOM/persistent OME used the denominator ‘children with POM’ as the denominator, the analyses for the outcome VTI surgery used the denominator ‘Children with referrals for POM’ as the denominator. Analyses were adjusted for the case-mix variables as described previously, with the addition of asthma and hay fever. From these models, we interpreted correlates of regional variation through the estimate for the preceding care step (ie, the confounder) which was reported as an OR with a 95% CI.

For those steps in the care pathway that showed a significant association with the outcomes, we determined whether these steps also affected regional variation by calculating the CV for these models.

### Ethics

The relevant governance bodies of Nivel-PCD approved the study under number NZR-00323.015. According to Dutch legislation, neither obtaining informed consent nor approval by a medical ethics committee is obligatory for this observational study containing no directly identifiable data.^36^,^38^

## Results

### Characteristics of study population

Six percent of the children had at least one GP contact for a new POM diagnosis, of which 5% had at least one GP contact for a new AOM diagnosis and 1% had at least one GP contact for a new OME diagnosis (table 2). Some differences were present in the baseline characteristics between the AOM and OME populations (table 2). Children aged 0–1 years comprised a smaller proportion of OME cases (4%) compared with AOM (8%). Gender distribution was balanced, with 49% female across groups. More children were living in a neighbourhood with a high SES (Q4: 33%–35%) compared with a low SES (Q1: 19%–22%). Similar urbanicity levels were found across the AOM, OME and POM populations, with most children having a medium to strong urbanicity level (categories 4 and 5). Comorbidities such as asthma (12%) and hay fever (4%–5%) were consistent across diagnoses. Referrals and VTI surgery were more frequent among OME patients than in the rest of the population (19% vs 7%). The percentage of children with persistent OME (10%) was higher than the percentage of children with frequent AOM (4%).

**Table 2:** Characteristics of patient population 2017 and 2018 (N/%)

	Children 0–12 years (272.790)	POM (15.727=100%)	AOM (13.117=100%)	OME (3.972=100%)
Age (years)				
0–1	8.614 (3%)	1.080 (7%)	1.033 (8%)	143 (4%)
2–3	37.847 (14%)	3.960 (25%)	3.536 (27%)	707 (18%)
4–5	45.119 (17%)	4.383 (28%)	3.644 (28%)	1.199 (30%)
6–12	181.210 (66%)	6.304 (40%)	4.904 (37%)	1.923 (48%)
Sex (female)	133.381 (49%)	7.659 (49%)	6.383 (49%)	1.954 (49%)
Neighbourhood SES*				
Q1 (lowest SES)	60.200 (22%)	3.393 (22%)	2.915 (22%)	755 (19%)
Q2	61.920 (23%)	3.683 (23%)	3.103 (24%)	914 (23%)
Q3	57.092 (21%)	3.309 (21%)	2.724 (21%)	894 (23%)
Q4 (highest SES)	93.578 (34%)	5.292 (34%)	4.335 (33%)	1.399 (35%)
Urbanicity				
1 (very strong urban; ≥2500 addresses/km^2^)	56.273 (21%)	3.101 (20%)	2.659 (20%)	675 (17%)
2 (strong urban; 1500–2500 addresses/km^2^)	69.296 (25%)	4.049 (26%)	3.399 (26%)	1.017 (26%)
3 (medium urban; 1000–1500 addresses/km^2^)	71.819 (26%)	4.284 (27%)	3.535 (27%)	1.127 (28%)
4 (scarcely urban; 500–1000 addresses/km^2^)	38.546 (14%)	2.214 (14%)	1.823 (14%)	603 (15%)
5 (non-urban; <500 addresses/km^2^)	36.856 (14%)	2.029 (13%)	1.661 (13%)	540 (14%)
Asthma	Data not available	1.877 (12%)	1.569 (12%)	487 (12%)
Hay fever	Data not available	695 (4%)	560 (4%)	200 (5%)
GP contact		15.727 (100%)	13.117 (100%)	3.972 (100%)
Frequent AOM/persistent OME†		921 (6%)	580 (4%)	341 (10%)
Referral		2.234 (14%)	1.533 (12%)	1.175 (30%)
VTI surgery		1349 (9%)	903 (7%)	758 (19%)

* Neighbourhood Socioeconomic Status (Dutch: SES-WOA): neighbourhood SES was measured using status scores indicating the status of a neighbourhood in comparison to other neighborhoods.^35^ The score is derived from several characteristics of individuals: education, income and position on the labour market and was divided into quartiles for analysis (a higher quartile indicating a higher SES).

† After ≥3 episodes in 6 months/≥4 episodes in 1 year for AOM and for OME after a care episode lasting ≥3 months.

AOM, acute otitis media; GP, general practitioner; OME, otitis media with effusion; POM, paediatric otitis media; SES, socioeconomic status; VTI, ventilation tube insertion.

### Degree of regional variation in POM treatment

**Figure 2:**
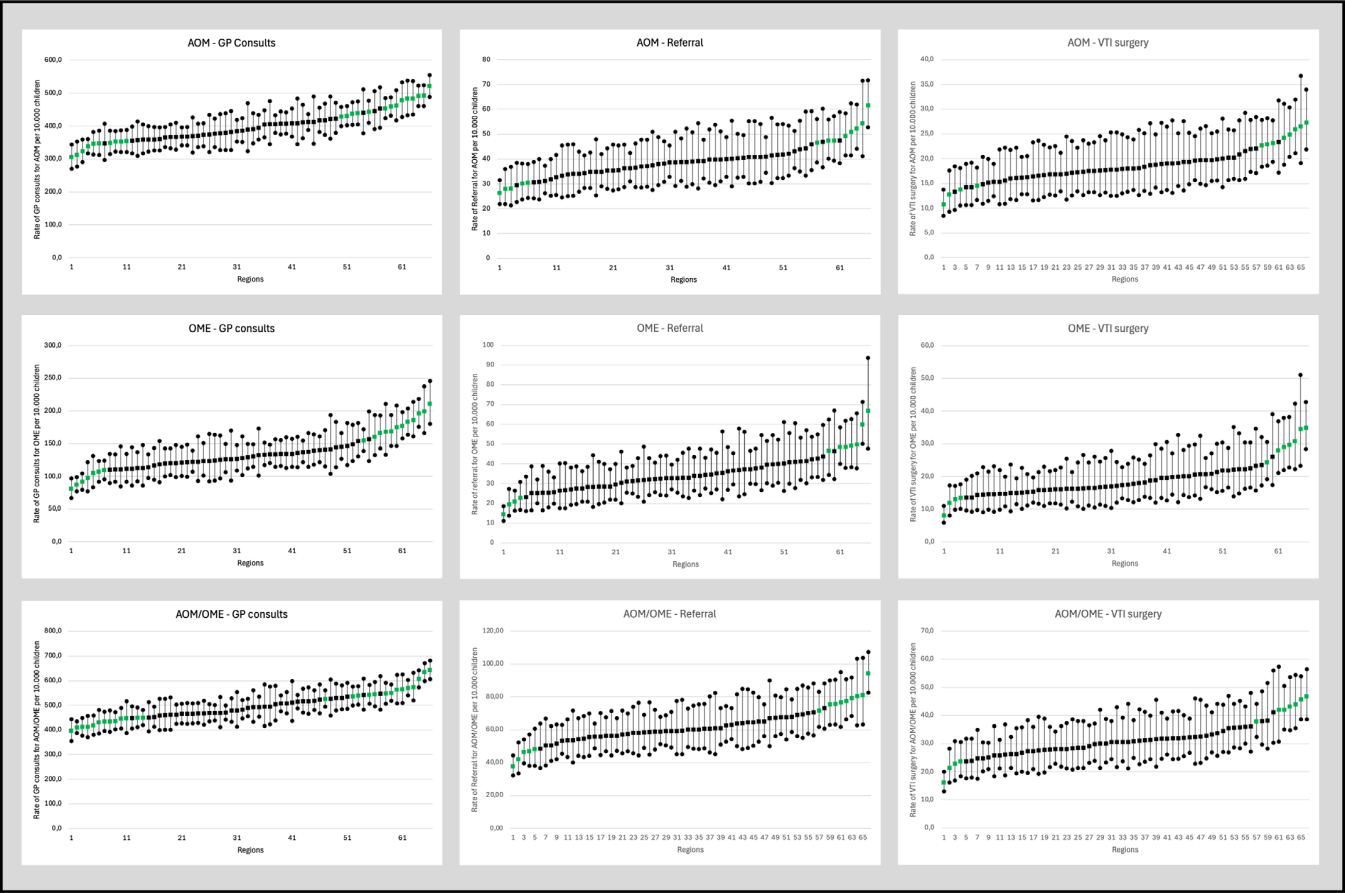
Adjusted regional rates of GP contacts, referrals and VTI surgery per 10 000 children for POM, AOM and OME population (adjusted for age, gender, neighbourhood SES and urbanicity). AOM, acute otitis media; GP, general practioner; OME, otitis media with effusion; POM, paediatric otitis media; SES, socioeconomic status; VTI, ventilation tube insertion.

For all populations—POM, AOM and OME—regional variation increased progressively with each subsequent step in the care pathway (table 3) (figure 2). Variation was lowest for GP contacts, increased for referrals and was highest for VTI surgery. Regional variation was most pronounced in the OME population, ranging from a CV of 0.197 for GP contacts to a CV of 0.271 for VTI surgery.

**Table 3:** Regional variation POM treatment

	Raw model	Adjusted model*
	CV	CV
POM		
GP contact	0.110	0.107
Referrals	0.179	0.162
VTI surgeries	0.239	0.192
AOM		
GP contact	0.118	0.117
Referrals	0.185	0.170
VTI surgeries	0.211	0.179
OME		
GP contact	0.207	0.197
Referrals	0.281	0.263
VTI surgeries	0.323	0.271

* Adjusted for age, gender, neighbourhood SES, urbanicity.

AOM, acute otitis media; GP, general practitioner; OME, otitis media with effusion; POM, paediatric otitis media; SES, socioeconomic status; VTI, ventilation tube insertion.

Adjusting for confounders (age, gender, neighbourhood SES and urbanicity) minimally impacted regional variation in GP contacts. While case-mix adjustments explained some of the variation in referrals and VTI surgeries, regional variation still increased with each subsequent step in the care pathways in the adjusted model.

Sensitivity analyses showed that the CV increased only slightly after excluding small regions, indicating that small regions did not significantly alter results.

### Adjustment for prior care steps when identifying treatment variation

Correlations between prior care steps and referrals to VTI surgeries are presented in table 4, with full model analyses detailed in online supplemental file 1.

**Table 4:** Correlations between sequential steps in the care pathway (n=66 regions)

		POM		AOM		OME	
		OR	95% CI	OR	95% CI	OR	95% CI
Individual probability of referral							
Preceding step	Included in model as follows:						
Patient-initiated GP contact	% of children per region with patient-initiated GP visit for POM	1.01	0.89 to 1.14	1.03	0.88 to 1.21	1.30	0.95 to 1.78
Frequent AOM/persistent OME†	% of children with patient-initiated GP contact for POM per region with frequent AOM/persistent OME	**1.06**	**1.02 to 1.11**	**1.11**	**1.04 to 1.19**	**1.04**	**1.00 to 1.08**
Individual probability of VTI surgery							
Patient-initiated GP contact	% of children per region with patient-initiated GP contact for POM	1.04	0.83 to 1.32	1.00	0.75 to 1.35	1.15	0.70 to 1.90
Referral*	% of children with patient-initiated GP contact for POM per region with referral	1.03	0.95 to 1.12	1.03	0.93 to 1.14	1.05	0.98 to 1.11
Frequent AOM/persistent OME†	% of children with patient-initiated GP contact for POM per region with frequent AOM/persistent OME	1.08	1.00 to 1.17	1.10	0.97 to 1.25	**1.08**	**1.02 to 1.15**

* Adjusted for confounders, % of children per region with patient-initiated GP contact and % of children with patient-initiated GP contact for POM per region with frequent AOM/persistent OME. Values in bold represent ORs with p-value <0.05

† After ≥3 episodes in 6 months/≥4 episodes in 1 year for AOM and for OME after a care episode lasting ≥3 months.

AOM, acute otitis media; GP, general practitioner; OME, otitis media with effusion; POM, paediatric otitis media; VTI, ventilation tube insertion.

For referrals, a higher regional level of GP contacts was associated with a higher likelihood of referrals in all patient populations, although the effect was not statistically significant. In the POM population, the OR was 1.01 (95% CI: 0.89 to 1.14), in the AOM population, the OR for GP contacts was 1.03 (95% CI: 0.88 to 1.21) and in the OME population, the OR was 1.30 (95% CI: 0.95 to 1.78).

A significant association was observed between frequent AOM/persistent OME and referrals: regions with a higher percentage of children with frequent AOM/persistent OME in GP practice showed significantly increased odds of referrals for all populations (denominator: children with patient-initiated GP contacts for POM). This association was stronger for AOM (OR: 1.11; 95% CI: 1.04 to 1.19) than for OME (OR: 1.04; 95% CI: 1.00 to 1.08). Correspondingly, regional variation in referrals decreased across all populations after adjusting for frequent AOM/persistent OME. For POM, regional variation declined by 20% when adjusted for frequent AOM compared with case-mix adjustment alone (CV 0.103 vs CV 0.128), for AOM, the reduction was 23% (CV 0.105 vs CV 0.136) and for OME, regional variation decreased by 11% (CV 0.081 vs CV 0.091).

For VTI surgery, a higher regional referral rate showed higher odds of surgery, though this association was not statistically significant in any population. In POM, the OR was 1.03 (95% CI: 0.95 to 1.12), in AOM, the OR was 1.03 (95% CI: 0.93 to 1.14) and for OME, the OR was 1.05 (95% CI: 0.98 to 1.11) (denominator: children with referral for POM).

A significant association was observed between persistent OME and VTI surgery: OR: 1.08 (95% CI: 1.02 to 1.15). Regional variation in VTI surgery for the OME population also decreased when adjusting for persistent OME compared with case-mix adjustment alone (CV 0.047 vs CV 0.059).

## Discussion

This study aimed to examine the degree of regional variation in each step of the POM care pathway and the relevance of adjusting for prior care processes when evaluating POM treatment variation, since treatment variation may be explained by prior care processes. Regional variation was observed in the whole care pathway and increased with each subsequent step in the care pathway. Positive correlations between subsequent steps were observed across the care pathway, although these were not significant between individual care steps. The only significant correlation of variation was the GP population with frequent AOM/persistent OME. Namely, regions with a higher percentage of children with frequent AOM/persistent OME in GP practice showed a higher odds of referral for all populations and a higher odds of VTI surgery for the OME population. Furthermore, while regional variation in referrals and VTI surgeries decreased after adjusting for frequent AOM/persistent OME, a substantial amount of treatment variation persisted.

The increased regional variation (CV) from patient-initiated GP contacts to referrals and VTI surgery may suggest that GP referrals and ENT surgical decision-making amplify variation of the previous care step. However, an increase in CV alone could result from factors beyond these referral patterns or surgical decisions. To identify whether GP referrals and ENT surgical decisions could explain the increase in CV, we examined the associations between sequential care steps. Interestingly, the association between sequential steps was positive across the care pathway, although associations were not significant between any of the individual steps. With the gatekeeper function of GPs, one could expect that the association between GP contacts and referrals is negative, as this would indicate a mitigation of patient-initiated variation in GP contacts. Namely, this would indicate that in regions with a higher portion of GP contacts than expected based on case-mix, GPs mitigate variation by not referring. However, this was not found in our study. Similarly, a negative association between referrals and VTI surgeries could be expected, which was not observed in our study either.

We did observe that regions with a higher degree of children with frequent AOM/persistent OME for referral in GP practice had higher odds of referrals. This suggests that GPs do partly adhere to the ‘appropriate referral’ part of their gatekeeping role.

### Comparison to existing literature

We found a limited number of studies that took into account variation in referral rates for POM, rather than focusing solely on surgical treatment variation. Literature is often focused on variation in antibiotic treatment, which is restricted in the Dutch guidelines, and therefore not the focus of this study.^39 40^

A study on POM treatment variation in Norway, which has a comparable gatekeeper system to the Netherlands, similarly found a moderate variation in referral patterns among GPs.^41^ They found that GPs with a completed specialty in General Medicine had reduced referrals for POM and that non-medical factors, such as GP workload, hospital access and availability of practising otolaryngologists were all determinants for referral. A study in Canada showed an almost 10-fold difference in surgical treatment between small areas, which is greater than the variation found in our study.^42^ This difference may be due to the chosen areas, as smaller areas tend to differ more than larger areas. A higher percentage of adults with at least completed high school education in an area and greater enthusiasm of referring physicians for surgery was associated with higher surgery level in an area, whereas the proportion of female referring physicians was associated with a lower level of surgical treatment in an area.

Furthermore, another Dutch study also examined the association between referral rates and surgical treatment for POM.^7^ This study was based on four hospital service areas within the same region and used 4-digit ZIP code to classify regions. They found a similar CV for VTI surgery for POM as our study. However, they found a markedly different VTI surgical rate (300 VTI surgeries/1000 inhabitants per hospital service area compared with 3.1 VTI surgeries/1000 inhabitants in our study). This could be explained as this study included only VTI surgery for children with a new POM, whereas Munster *et al* included all VTI surgeries independent of the registered diagnosis. In addition, Munster *et al* included a broader group of diagnoses and patients up to 15 years old. Regarding the association between referral rates and VTI surgeries, Munster *et al* found that referral rates were associated with 2-digit ZIP code surgical rates and explained approximately 49% (r=0.70, r^2^=0.49, p=0.008) of the regional variation, but referral rates were not associated with in-hospital surgical rates (r=−0.41, r^2^=0.18, p=0.15).

This study found much lower variation in VTI surgeries than in other countries.^6 43 44^ Perhaps this is due to the availability of Dutch clinical guidelines for both GPs and ENTs, the national attention towards conservative management for POM and the GP gatekeeper function. This confirms the common principle that when there is agreement on indications for surgery, treatment variation tends to be low.^45^ Larger treatment variation was observed in the OME population than in the AOM population. This could be explained as Dutch clinical guidelines for OME offer less specific recommendations for diagnosis and treatment than AOM guidelines.

### Implications

Negative associations across the care pathway would indicate a mitigating role of GPs and ENT specialists in reducing patient-initiated variation, but this was not observed. Rather, our results indicate that regions with an over-representation in GP contacts rate have an over-representation of referrals and VTI surgeries, while regions with an under-representation in GP contacts rate have an under-representation in referrals and VTI surgeries. This suggests that in regions, patients, GPs and specialists’ actions are not independent, but clinical decision making at one level impacts clinical decision making at other levels. Specifically, when specialists perform surgeries following referral, this gives a feedback loop to both patients/parents and GPs that referral was warranted, even if this may not be the case based on patient characteristics and disease severity. As all stakeholders (patients GPs and ENT specialists) can independently increase or mitigate unwarranted treatment variation, it is valuable to target each stakeholder in their role. While A&F interventions can effectively reduce unwarranted treatment variation, they only work when feedback is actionable for the person who can directly influence the behaviour.^10 12^ Thus, to reduce unwarranted variation in VTI surgeries, it is valuable to provide A&F which considers the impact of prior care steps on that variation and to target both specialists and GPs.

For future research, it is interesting to examine whether the observed pattern of increasing variation across healthcare steps, suggesting a feedback loop between specialists, general practitioners and patients, is unique to POM or represents a broader phenomenon common to other areas of healthcare practice.

### Strengths and limitations

This study’s primary strength lies in its examination of treatment variation and correlations of subsequent steps in the care pathway, rather than focussing on variation in specialist care. Furthermore, this study benefits from patient-level data of a representative sample of the Dutch population. The ability to track patients from general practice into secondary care over an extended period enhances the robustness of our findings. Additionally, focusing on a healthcare system with a gatekeeper function provides valuable insights for policymakers aiming to reduce treatment variation.

An important limitation is this study’s use of registry and claims data, which lack detailed information on disease severity and the subjective rationale behind treatment decisions. This constraint hinders our ability to fully distinguish between overtreatment and undertreatment within the data set. Furthermore, we examined variation on the regional level. At regional level, patient populations are more similar, thus regional treatment variation is more likely to be unwarranted than at the individual provider treatment variation. However, patient populations are only considered comparable if regions are large enough and adjustment for confounding effects has been made. In this study, we used hospital service areas, which may not have been sufficiently large areas to rule out population differences entirely. Finally, to gain a deeper understanding of the role of GPs versus specialists, it can be valuable to conduct treatment variation research of the level of GP practices and hospitals, rather than regions. However, to rule out warranted variation due to differences in patient need at the unique provider level, more detailed data regarding disease severity would be required in this case.

## Conclusion

Regional variation is observed in GP contacts for POM, which persists and seems to be amplified in later stages of the care pathway: in referrals and VTI surgery. The absence of negative associations between sequential care steps suggests that GP referrals and ENT surgical decisions do not mitigate this patient-initiated variation. Additionally, regional variation in referrals for all populations and OME-related VTI surgeries decreased after adjusting for frequent AOM/persistent OME. Consequently, quality improvement initiatives could benefit from addressing the input and management of all stakeholders in the care pathway, rather than focussing solely on specialists. Furthermore, A&F can provide a more accurate reflection of each stakeholder’s role when it is adjusted for the prior care steps. Thus, future A&F aiming to reduce surgical treatment variation should consider the care pathway to develop tailored interventions for quality improvement.

## Supplementary material

10.1136/bmjopen-2025-101692 online supplemental file 1

## Data Availability

Data may be obtained from a third party and are not publicly available.
