# Supplementary material for: Considering the full care pathway in regional variation in paediatric otitis media treatment in the Netherlands: an observational study
Source: BMJ Open. 2025 Aug 19;15(8):e101692. doi: 10.1136/bmjopen-2025-101692 (PMC12366597; doi:10.1136/bmjopen-2025-101692)
Supplement: online supplemental file 1 [file bmjopen-15-8-s001.docx]

# Supplemental File 1 | Full Models

**Table. Referral – POM population**

*Correlation GP contacts and Frequent AOM/persistent OME respectively with Referral*

|  | **OR** | **95%CI** |
| --- | --- | --- |
| Age group (reference=age 0-1 year) |  |  |
| 2-3 years | 1.09 | 0.9-1.32 |
| 4-5 years | 1.38 | 1.14-1.67 |
| 6-12 years | 0.72 | 0.59-0.87 |
| Gender | 0.83 | 0.76-0.91 |
| Neighbourhood SES (reference=quartile 1 – lowest) |  |  |
| Quartile 2 | 1.15 | 0.99-1.34 |
| Quartile 3 | 1.06 | 0.9-1.25 |
| Quartile 4 | 0.99 | 0.86-1.15 |
| Urbanicity level (reference=extremely urbanised) |  |  |
| Strongly urbanised | 1.25 | 1.07-1.45 |
| Moderately urbanised | 1.34 | 1.14-1.58 |
| Hardly urbanised | 1.21 | 1-1.46 |
| Not urbanised | 1.35 | 1.11-1.64 |
| Asthma | 1.03 | 0.9-1.19 |
| Hay Fever | 1.28 | 1.03-1.59 |
| **GP contacts** | **1.01** | 0.89-1.14 |
| **Frequent AOM/persistent OME** | **1.06** | 1.02-1.11 |

**Table. Referral – AOM population**

*Correlation GP contacts and Frequent AOM/persistent OME respectively with Referral*

|  | **OR** | **95%CI** |
| --- | --- | --- |
| Age group (reference=age 0-1 year) |  |  |
| 2-3 years | 0.93 | 0.76-1.14 |
| 4-5 years | 1.07 | 0.88-1.31 |
| 6-12 years | 0.56 | 0.46-0.69 |
| Gender | 0.80 | 0.72-0.89 |
| Neighbourhood SES (reference=quartile 1 – lowest) |  |  |
| Quartile 2 | 1.16 | 0.97-1.38 |
| Quartile 3 | 1.08 | 0.89-1.31 |
| Quartile 4 | 0.99 | 0.83-1.18 |
| Urbanicity level (reference=extremely urbanised) |  |  |
| Strongly urbanised | 1.27 | 1.06-1.52 |
| Moderately urbanised | 1.34 | 1.11-1.63 |
| Hardly urbanised | 1.24 | 0.99-1.55 |
| Not urbanised | 1.34 | 1.07-1.69 |
| Asthma | 1.01 | 0.85-1.19 |
| Hay Fever | 1.19 | 0.91-1.56 |
| **GP contacts** | **1.03** | **0.88-1.21** |
| **Frequent AOM/persistent OME** | **1.11** | **1.04-1.19** |

**Table. Referral – OME population**

*Correlation GP contacts and Frequent AOM/persistent OME respectively with Referral*

|  | **OR** | **95%CI** |
| --- | --- | --- |
| Age group (reference=age 0-1 year) |  |  |
| 2-3 years | 1.08 | 0.74-1.58 |
| 4-5 years | 1.23 | 0.85-1.78 |
| 6-12 years | 0.49 | 0.34-0.71 |
| Gender | 0.83 | 0.72-0.96 |
| Neighbourhood SES (reference=quartile 1 – lowest) |  |  |
| Quartile 2 | 0.93 | 0.74-1.18 |
| Quartile 3 | 0.83 | 0.65-1.07 |
| Quartile 4 | 0.78 | 0.62-0.97 |
| Urbanicity level (reference=extremely urbanised) |  |  |
| Strongly urbanised | 1.26 | 1.00-1.60 |
| Moderately urbanised | 1.35 | 1.06-1.73 |
| Hardly urbanised | 1.32 | 0.99-1.75 |
| Not urbanised | 1.58 | 1.18-2.11 |
| Asthma | 0.97 | 0.78-1.21 |
| Hay Fever | 1.19 | 0.86-1.65 |
| **GP contacts** | **1.30** | **0.95-1.78** |
| **Frequent AOM/persistent OME** | **1.04** | **1.00-1.08** |

**Table. VTI surgery – POM population**

*Correlation GP contacts, Referral and Frequent AOM/persistent OME respectively with VTI surgery*

|  | **OR** | **95%CI** |
| --- | --- | --- |
| Age group (reference=age 0-1 year) |  |  |
| 2-3 years | 0.77 | 0.52-1.14 |
| 4-5 years | 0.59 | 0.41-0.87 |
| 6-12 years | 0.29 | 0.20-0.43 |
| Gender | 0.98 | 0.82-1.17 |
| Neighbourhood SES (reference=quartile 1 – lowest) |  |  |
| Quartile 2 | 0.94 | 0.70-1.25 |
| Quartile 3 | 0.96 | 0.70-1.32 |
| Quartile 4 | 1.02 | 0.77-1.36 |
| Urbanicity level (reference=extremely urbanised) |  |  |
| Strongly urbanised | 1.29 | 0.96-1.73 |
| Moderately urbanised | 1.63 | 1.19-2.22 |
| Hardly urbanised | 1.01 | 0.70-1.45 |
| Not urbanised | 1.42 | 0.98-2.07 |
| Asthma | 0.98 | 0.75-1.28 |
| Hay Fever | 0.74 | 0.49-1.11 |
| **GP contacts** | **1.04** | **0.83-1.32** |
| **Referral** | **1.03** | **0.95-1.12** |
| **Frequent AOM/persistent OME** | **1.08** | **1.00-1.17** |

**Table. VTI surgery – AOM population**

*Correlation GP contacts, Referral and Frequent AOM/persistent OME respectively with VTI surgery*

|  | **OR** | **95%CI** |
| --- | --- | --- |
| Age group (reference=age 0-1 year) |  |  |
| 2-3 years | 0.76 | 0.51-1.14 |
| 4-5 years | 0.62 | 0.42-0.93 |
| 6-12 years | 0.29 | 0.19-0.43 |
| Gender | 0.99 | 0.80-1.23 |
| Neighbourhood SES (reference=quartile 1 – lowest) |  |  |
| Quartile 2 | 0.86 | 0.61-1.21 |
| Quartile 3 | 0.87 | 0.59-1.27 |
| Quartile 4 | 0.92 | 0.65-1.29 |
| Urbanicity level (reference=extremely urbanised) |  |  |
| Strongly urbanised | 1.10 | 0.78-1.56 |
| Moderately urbanised | 1.71 | 1.17-2.49 |
| Hardly urbanised | 1.10 | 0.71-1.69 |
| Not urbanised | 1.41 | 0.90-2.20 |
| Asthma | 0.88 | 0.64-1.22 |
| Hay Fever | 0.75 | 0.45-1.26 |
| **GP contacts** | **1.00** | **0.75-1.35** |
| **Referral** | **1.03** | **0.93-1.14** |
| **Frequent AOM/persistent OME** | **1.10** | **0.97-1.25** |

**Table. VTI surgery – OME population**

*Correlation GP contacts, Referral and Frequent AOM/persistent OME respectively with VTI surgery*

|  | **OR** | **95%CI** |
| --- | --- | --- |
| Age group (reference=age 0-1 year) |  |  |
| 2-3 years | 0.66 | 0.31-1.41 |
| 4-5 years | 0.41 | 0.20-0.86 |
| 6-12 years | 0.18 | 0.09-0.38 |
| Gender | 0.94 | 0.73-1.20 |
| Neighbourhood SES (reference=quartile 1 – lowest) |  |  |
| Quartile 2 | 1.23 | 0.83-1.81 |
| Quartile 3 | 1.25 | 0.82-1.90 |
| Quartile 4 | 1.29 | 0.88-1.91 |
| Urbanicity level (reference=extremely urbanised) |  |  |
| Strongly urbanised | 1.97 | 1.30-2.99 |
| Moderately urbanised | 1.67 | 1.09-2.56 |
| Hardly urbanised | 1.10 | 0.67-1.81 |
| Not urbanised | 1.66 | 1.00-2.76 |
| Asthma | 1.11 | 0.76-1.63 |
| Hay Fever | 0.84 | 0.48-1.48 |
| **GP contacts** | **1.15** | **0.70-1.90** |
| **Referral** | **1.05** | **0.98-1.11** |
| **Frequent AOM/persistent OME** | **1.08** | **1.02-1.15** |
