# Supplementary material for: Considering the full care pathway in regional variation in paediatric otitis media treatment in the Netherlands: an observational study
Source: BMJ Open. 2025 Aug 19;15(8):e101692. doi: 10.1136/bmjopen-2025-101692 (PMC12366597; doi:10.1136/bmjopen-2025-101692)
Supplement: Supplementary file 2 [file bmjopen-2025-101692.reviewer_comments.pdf]

PEER REVIEW HISTORY

BMJ Open publishes all reviews undertaken for accepted manuscripts. Reviewers are asked to complete a checklist review form and are provided with free text boxes to elaborate on their assessment. These free text comments are reproduced below.

ARTICLE DETAILS

Title (Provisional)

Considering the full care pathway in regional variation in paediatric otitis media treatment in the Netherlands: an observational study.

Authors

de Weerdt, Vera; van Dijk, Christel; Burgers, Jako; Gaspar, Kati; Hek, Karin; Hemler, Raphael J; Repping, S.; Verheij, Robert A; Willems, Hanna; van der Hijden, Eric; Koolman, Xander

---

VERSION 1 - REVIEW

**Reviewer**	**1**
**Name**	**Ikeda, Ryoukichi**
**Affiliation**	**Tohoku University Graduate School of Medicine**
**Date**	**02-May-2025**
**COI**	**None**

Thank you for this highly interesting and well-conducted study. The manuscript addresses an important clinical topic. I would like to offer the following suggestions to further enhance the clarity and impact of the work:

Since the present study is based on data from the Netherlands, it may be helpful to provide a brief overview of the characteristics of Dutch clinical guidelines for AOM and OME. Guidelines may vary significantly between countries, and including this context would assist international readers in interpreting the findings more accurately.

The section discussing OME and tympanostomy tube placement is clearly presented and easy to follow. However, the rationale for tube insertion in cases of AOM is less clear. In many countries, tympanostomy tubes are typically considered for recurrent AOM, rather than isolated episodes. To improve clarity, it may be helpful to either focus solely on OME or, alternatively, explain the Dutch clinical approach to AOM management and how it may differ from other national guidelines.

**Reviewer**	**2**

Name Marom, T

Affiliation Assuta Ashdod University Hospital, Otolaryngology-Head and Neck Surgery

Date 10-May-2025

COI None

Nice paper, trying to reach the roots of regional variation for the treatment of pediatric otitis media in the Netherlands. Unfortunately, too many overstatements across the manuscript.

Several comments/edits that need to be addressed.

The abstract should be revised. What gap in the literature or evidence, even from your own country, is there, why is it important, and how will you bridge it? The research goal is not very clear. Coming from the clinical world, I can't understand your findings and their importance. I am sorry.

Introduction. "In the past, this surgery was signaled for potential unwarranted treatment variations, and it became a topic of global deimplementation initiatives" - this is not true. You need to tone it down. I understand that parents are partners in the decisions, but from your text, it seems that they are the ones who decide and not health professionals.

Methods. What were the parameters that you extracted? Overall, it was very long this section and hard to follow. You need to trim it by at least 30%.

Results. Need to be better organized. "The minimum number of patients met OME per region was 3, maximum number 198, mean 59" - if the regions were not equal (as I expect them not to be), what is the significance? From your tables, it seems that children aged 6-12 years have the highest burden of otitis media. Well, that can not be true, as the younger children are the ones to suffer from OM. Double-check your numbers. Again, it was very hard to follow all your results. Consider preparing online tables as supplements.

Discussion. OK and thorough. Omit overstatements.

References should include more recent OM studies.

VERSION 1 - AUTHOR RESPONSE

Reviewer 1: Ryoukichi Ikeda, Tohoku University Graduate School of Medicine

1. Since the present study is based on data from the Netherlands, it may be helpful to provide a brief overview of the characteristics of Dutch clinical guidelines for AOM and OME. Guidelines may vary significantly between countries, and including this context would assist international readers in interpreting the findings more accurately.

We understand that it is helpful for international readers to have more information about the Dutch clinical guidelines. In the revised manuscript we added a text box with information on the Dutch setting and clinical guidelines.

2. The section discussing OME and tympanostomy tube placement is clearly presented and easy to follow. However, the rationale for tube insertion in cases of AOM is less clear. In many countries, tympanostomy tubes are typically considered for recurrent AOM, rather than isolated episodes. To improve clarity, it may be helpful to either focus solely on OME or, alternatively, explain the Dutch clinical approach to AOM management and how it may differ from other national guidelines.

   Dutch ENT guidelines recommend considering surgical treatment with Ventilation tube insertion for frequently recurring AOM, following the same definition of GPs (≥3 episodes within 6 months or ≥4 episodes within 1 year. This was added in a box in the revised manuscript.

Reviewer 2: Dr. T Marom, Assuta Ashdod University Hospital

3. The abstract should be revised. What gap in the literature or evidence, even from your own country, is there, why is it important, and how will you bridge it? The research goal is not very clear. Coming from the clinical world, I can't understand your findings and their importance. I am sorry.

   We agree that the gap in the literature was stated rather indirectly. In the revised manuscript we adjusted the abstract:

   *"**Objective:** Ventilation tube insertion for paediatric otitis media (POM), including acute otitis media (AOM) and otitis media with effusion (OME), has been signalled in the past for potential unwarranted treatment variation. Quality improvement initiatives, like Audit & Feedback (A&F), often ignore the care pathway when identifying such variation, possibly overestimating variation at a specific care step. To gain more insight into the effect of prior care steps, this study examined (1) the degree of regional variation in each step of the care pathway (GP contacts, referrals and surgeries) and (2) investigated the effect of adjusting for prior care steps."*

4. Introduction. "In the past, this surgery was signaled for potential unwarranted treatment variations, and it became a topic of global deimplementation initiatives" - this is not true. You need to tone it down.

   In the revised manuscript we excluded the word "global" to prevent overstatement.

5. I understand that parents are partners in the decisions, but from your text, it seems that they are the ones who decide and not health professionals.

We agree that health professionals decide together with parents about the treatment. In the manuscript, we now state that patients (or parents), GPs and ENT specialist play a role in the care pathway.

10 Methods. What were the parameters that you extracted? Overall, it was very long this section and hard to follow. You need to trim it by at least 30%.

In the revised manuscript, we reorganized the methods section into (1) Region, (2) Study outcome: regional variation, (3) Factors possibly explaining regional variation, and (4) Potential confounders, in order to present the extracted parameters in a more organized way. We trimmed the Methods. The Methods section is still extensive. We believe that this is necessary to understand the manuscript.

11 Results: Need to be better organized.

In the revised manuscript, we revised the organization of the results. Some parts were moved to the Methods: (1) description of number of included patients and GP practices, and (2) description of the regions. Furthermore, figure 2 was moved to the section "Degree of regional variation in POM treatment".

12 Results: The minimum number of patients met OME per region was 3, maximum number 198, mean 59" - if the regions were not equal (as I expect them not to be), what is the significance?

This part of the Results section was moved to the Methods section. When assessing regional variation, it is important to know the number of patients/children per region. In regions with a lower number of patients, the degree of certainty is lower. In our study, some regions had a lower number of patients per region, but overall, the mean number of patients per region was substantial.

13 Results: From your tables, it seems that children aged 6-12 years have the highest burden of otitis media. Well, that can not be true, as the younger children are the ones to suffer from OM. Double-check your numbers. Again, it was very hard to follow all your results. Consider preparing online tables as supplements.

It is true that younger children suffer more from POM. Table 2 shows the percentage of children with POM within a certain age category. 40% of the children are aged 6-12 years. It may seem that older children suffer more, however the this age category is broader than the younger age categories. 2.5% of the children aged 0-1 year suffered from POM (1,080/8,614), 10.5% of the children aged 2-3 years of age (3,960/37,847), 9.7% of the children aged 4-5 years of age (4,383/45,119) and 3.5% of the children aged 6-12 years of age (6,304/181,210).

14 Discussion. OK and thorough. Omit overstatements.

The discussion was revised to omit overstatements. We deleted the following sentence:
"Their finding that the availability of practicing otalaryngologists increases the likelihood of referral correspond with the correlation found in our study, suggesting that GPs and ENT specialists' decision making are influenced by each other and not independent"
We changed the last sentence of the implications:
"For future research, it is interesting to examine whether the observed pattern of increasing variation across healthcare steps, suggesting a feedback loop between specialists, general practitioners, and patients, is unique to POM or represents a broader phenomenon common to other areas of healthcare practice."

15 References should include more recent OM studies.

In the revised manuscript, we included more recent OM studies. However, most recent studies in treatment variation in POM focus on antibiotic treatment. We included the following statement:

"Literature is often focussed on variation in antibiotic treatment, which is restricted in the Dutch guidelines, and therefore not the focus of this study"

And added a study on variation in surgical treatment from Canada.

VERSION 2 - REVIEW

**Reviewer**	**2**
**Name**	**Marom, T**
**Affiliation**	**Assuta Ashdod University Hospital, Otolaryngology-Head and Neck Surgery**
**Date**	**08-Jul-2025**
**COI**	

Well done. This is a much better manuscript. Congratulations!
